# Revised cardiac risk index in predicting cardiovascular complications in patients receiving chronic kidney replacement therapy undergoing elective general surgery

**DOI:** 10.1186/s13741-024-00429-0

**Published:** 2024-07-10

**Authors:** Dharmenaan Palamuthusingam, Elaine M. Pascoe, Carmel M. Hawley, David Wayne Johnson, Magid Fahim

**Affiliations:** 1 Metro North Kidney Service, Royal Brisbane and Women’s Hospital, Butterfield Street, Herston, QLD Australia; 2 Faculty of Medicine, University of Queensland, St Lucia, QLD 4072 Australia; 3 School of Medicine, Griffith University, Southport, QLD Australia; 4 Centre for Health Services Research, University of Queensland, St Lucia, QLD Australia; 5 Metro South Kidney and Transplant Services, Princess Alexandra Hospital, 199 Ipswich Road, Woolloongabba, QLD 4074 Australia; 6 Australasian Kidney Trials Network (AKTN), University of Queensland, St Lucia, QLD Australia; 7 Translational Research Institute, Brisbane, Australia; 8 Metro North Health Service, Butterfield Street, Herston, QLD Australia

**Keywords:** Perioperative medicine, Perioperative risk, Dialysis, Cardiovascular disease, Kidney transplant, Postoperative outcomes

## Abstract

**Introduction:**

The Revised Cardiac Risk Index (RCRI) is a six-parameter model that is commonly used in assessing individual 30-day perioperative cardiovascular risk before general surgery, but its use in patients on chronic kidney replacement therapy (KRT) is unvalidated. This study aimed to externally validate RCRI in this patient group over a 15-year period.

**Methods:**

Data linkage was used between the the Australia and New Zealand Dialysis and Transplant (ANZDATA) Registry and jurisdictional hospital admisisons data across Australia and New Zealand to identify all incident and prevalent patients on chronic KRT between 2000 and 2015 who underwent elective abdominal surgery. Chronic KRT was categorised as haemodialysis (HD), peritoneal dialysis (PD), home haemodialysis (HHD) and kidney transplant. The outcome of interest was major adverse cardiovascular event (MACE) which was defined as nonfatal myocardial infarction, nonfatal stroke, non-fatal cardiac arrest and cardiovascular mortality at 30 days. Logistic regression was used with the RCRI score included as a continuous variable to estimate discrimination by area under the receiver operating curve (AUROC). Calibration was evaluated using a calibration plot. Clinical utility was assessed using a decision curve analysis to determine the net benefit.

**Results:**

A total of 5094 elective surgeries were undertaken, and MACE occurred in 153 individuals (3.0%). Overall, RCRI had poor discrimination in patients on chronic KRT undergoing elective surgery (*AUROC* 0.67), particularly in patients aged greater than 65 years (*AUROC* 0.591). A calibration plot showed that RCRI overestimated risk of MACE. The expected-to-observed outcome ratio was 6.0, 5.1 and 2.5 for those with RCRI scores of 1, 2 and ≥ 3, respectively. Discrimination was moderate in patients under 65 years and in kidney transplant recipients, with AUROC values of 0.740 and 0.718, respectively. Overestimation was common but less so for kidney transplant recipients. Decision curve analysis showed that there was no net benefit of using the tool in neither the overall cohort nor patients under 65 years, but a slight benefit associated with threshold probability > 5.5% in kidney transplant recipients.

**Conclusions:**

The RCRI tool performed poorly and overestimated risk in patients on chronic dialysis, potentially misinforming patients and clinicians about the risk of elective surgery. Further research is needed to define a more comprehensive means of estimating risk in this unique population.

**Supplementary Information:**

The online version contains supplementary material available at 10.1186/s13741-024-00429-0.

## Introduction

Patients on chronic kidney replacement therapy (KRT) are considered to have a more than threefold higher odds for cardiovascular complications compared to the general population (Palamuthusingam et al. 2020; Palamuthusingam et al. 2021). Therefore, accurate and objective perioperative risk assessment is necessary not only to facilitate shared decision-making between clinicians and patients about the benefits and risk of surgery, but also to inform resource allocation and health service planning. Existing perioperative risk assessment tools used to predict perioperative cardiovascular risk remain unvalidated in patients on chronic KRT and, as such, may lead to these patients being labelled ‘high-risk’ surgical candidates, thereby potentially adversely impacting their access to surgery (Palamuthusingam et al. 2019).

The Revised Cardiac Risk Index (RCRI) is one of the commonly used tools for assessing individual 30-day perioperative risk of a major adverse cardiovascular event (MACE), defined as nonfatal myocardial infarction, nonfatal stroke and cardiovascular mortality, following elective surgery (Lee et al. 1999). The RCRI scores 1 point each for a history of ischemic heart disease, cerebrovascular disease, heart failure, elevated serum creatinine (> 177 µmol/L), insulin requiring diabetes mellitus and high-risk surgery (thoracic or intra-abdominal), with a higher total score being associated with an increased risk of MACE (Lee et al. 1999). In the general population, it has been shown to be moderately good at discriminating patients who will develop cardiac complications from those that will not following major noncardiac surgery (Barnett and Moonesinghe 2011). In fact, the European Society of Cardiology guidelines on perioperative risk assessment and the American College of Cardiology/American Heart Association guidelines endorse its use (Members et al. 2009; Smilowitz and Berger 2020). The RCRI tool risk stratifies patients, facilitating further cardiac assessment and optimisation, adoption of risk mitigation strategies in the perioperative setting or dissuading clinicians and patients from undergoing prohibitively high-risk surgery (Fleisher et al. 2007; Kristensen et al. 2014). A single North American study attempted to validate the RCRI in patients with kidney failure undergoing surgery and showed that this tool performed poorly. However, this study also included patients not requiring dialysis and evaluated a broad range of surgical interventions (Harrison et al. 2022). As such, the validity of its use in patients receiving chronic KRT remains unknown. This study aimed to evaluate the utility of the RCRI in patients receiving chronic KRT undergoing elective abdominal surgery.

## Methods

### Data sources

Data linkage between the Australia and New Zealand Dialysis and Transplant (ANZDATA) Registry and hospital admission datasets across all jurisdictions was used to identify all incident and prevalent patients aged ≥ 18 years receiving chronic KRT who underwent abdominal surgery between January 2000 and December 2015 with a length of stay greater than 2 days. ANZDATA registered all patients in Australia and New Zealand receiving chronic KRT. KRT was defined as patient receiving facility haemodialysis (HD), peritoneal dialysis (PD), home haemodialysis (HHD) or having a functioning kidney transplant. Jurisdictional hospital admission datasets recorded all demographic and clinical information following an individual’s hospital admission. This included all public and private hospital hospitals, except for Tasmania where only public hospital admissions data were stored. Clinical information on complications and comorbidities were coded as per the International Classification of Diseases 10th revision Australian modification (ICD-10-AM), whilst all surgeries and interventions undergone during the hospital admission were defined by the Australian Classification of Health Interventions (ACHI).

### Data linkage

Probabilistic linkage, using a combination of unique personal identifiers, such as first name, last name, date of birth, sex, address, and hospital identification number, was utilised to generate the likelihood that a hospital separation was associated with an individual. This was undertaken by each jurisdiction’s dedicated data linkage unit: New South Wales Admitted Patient Data Collection (APDC) linkage was undertaken by the Centre for Health Record Linkage (CHeReL), Queensland Hospital Admitted Patient Data Collection (QHAPDC) by the Statistical Services Branch of Queensland Health, South Australian Inpatient Hospital Separations and Northern Territory Inpatient Activity data set by South Australia and Northern Territory (SANT) DataLink, Tasmania Public Hospital Admitted Patient Collection by the Tasmanian Data Linkage Unit (TDLU), Victorian Admitted Episodes Datasets (VAED) by the Centre of Victorian Data Linkage (CVDL) and Western Australia Hospital Morbidity Data Collection by the WA Research Data Services. In New Zealand, deterministic linkage was used to link individual hospital records to each person using the National Health Index number (NHI) by the Ministry of Health in New Zealand.

### Covariates

Demographic data, including ethnicity, sex and age, as well as relevant KRT treatment details such as cause of kidney failure, dialysis modality, vintage, access, immunosuppression use, kidney transplant function and date and cause of death, were extracted from ANZDATA. Comorbid conditions and MACE events were obtained using an appropriate lookback period (Palamuthusingam, et al. 2020). All iterations of the ICD-10-AM codes are shown in Supplementary Table 1. ACHI codes identifying the types of abdominal surgery are provided in Supplementary Table 2.

### Outcomes

The outcome measure was a composite of nonfatal MI, nonfatal stroke, nonfatal cardiac arrest and 30-day cardiovascular mortality, herein referred to as MACE. ICD-10-AM codes used are provided in Supplementary Table 3.

### Statistics

The first elective abdominal surgery that each person underwent was used in the analyses. Patient characteristics were described using medians and interquartile ranges (IQR) for continuous variables and counts and percentages for categorical variables. Multivariable logistic regression with each of the RCRI variables and MACE was performed. In accordance with the original paper, these characteristics were then grouped by their RCRI classes (0 point = class 1, 1 point = class II, 2 point class III, ≥ 3 points = class IV) (Lee et al. 1999). Reference event rates were calculated from the original RCRI cohort (Lee et al. 1999). Patients with a score of 0 were assigned a 3.9% probability of MACE, 6.0% for patients with a score of 2, and 15% for those with a score of 3 or more. Logistic regression was used with RCRI score included as a continuous variable to estimate discrimination by area under the receiver operating curve (AUROC). An AUROC under 0.7 indicated poor performance, 0.7–0.9 was moderate performance, and greater than 0.9 was considered high performance (Swets 1988). The analyses were repeated for subgroups defined by age (< 65 and ≥ 65 years) and KRT modality (chronic dialysis and kidney transplant recipients). Calibration was evaluated using a calibration plot. Clinical utility was assessed using a decision curve analysis to determine the net benefit (NB) (Vickers et al. 2016). This required establishing threshold probabilities at which using the model may be beneficial for clinical decision-making. These probabilities were based on the weighted difference between true positives and false positives (Vickers et al. 2016). In this analysis, a clinically reasonable range was a probability of greater than 5%, meaning that patients with more than 5% risk from the model may have warranted high-dependency monitoring (HDU) and proactive cardiac biomarker testing. Secondary analyses were undertaken for patients receiving chronic dialysis and kidney transplant recipients separately. Exploratory analysis evaluating patients undergoing emergency abdominal surgery was also conducted.

## Ethics approval

Ethics approval for this study was provided by human research ethics committees in each of the health jurisdictions involved: New South Wales (HREC/17/CIPHS/41), Queensland (HREC/17/QPAH/636), South Australia (HREC/17/SAH/115), South Australia Aboriginal Health and Research Council (HREC 04–17-746), Northern Territory (HREC: 2017–2962), Department of Health Western Australia (RGS0000000740), Western Australia Aboriginal Health Ethics Committee (HREC: 835), Victoria (HREC/17/QPAH/638 — Victoria Specific Module under National Mutual Acceptance memorandum) and Tasmania (H0017537).

## Results

**Fig. 1:**
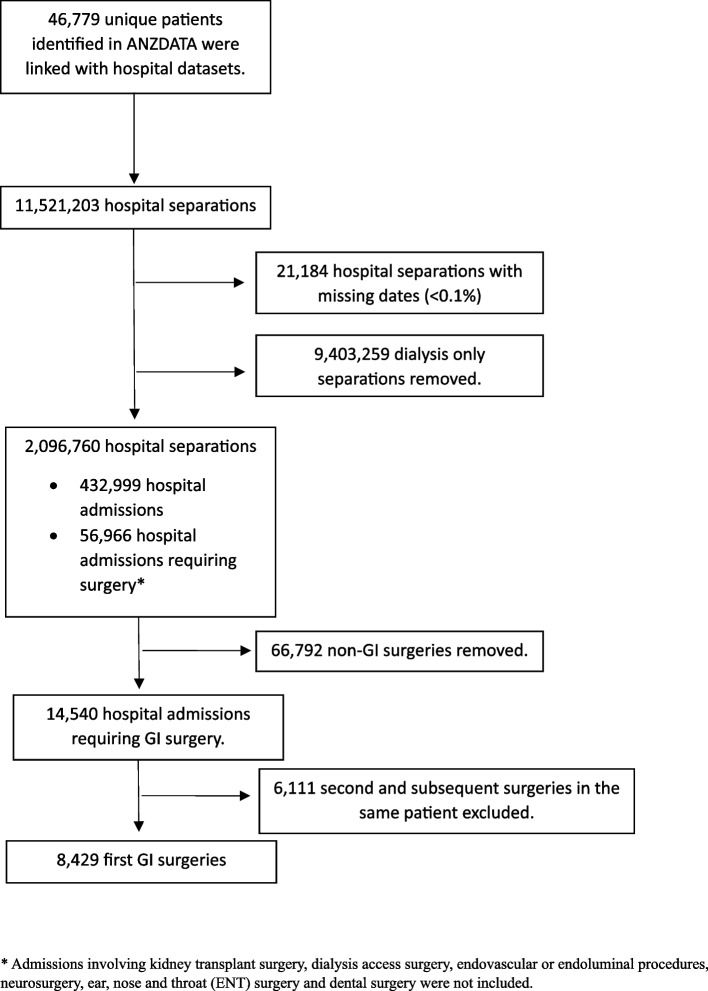
Cohort flow diagram. *Admissions involving kidney transplant surgery, dialysis access surgery, endovascular or endoluminal procedures, neurosurgery, ear, nose, and throat (ENT) surgery and dental surgery were not included

A total of 8429 first abdominal surgeries was identified, of which 5094 were elective procedures (Fig. 1). Abdominal surgery was classified as high-risk surgery, and therefore, no patients had a score of 0. Approximately, 38% of patients had a score of 3 or more. Older age, higher percentage of males and greater comorbidity burden were associated with higher RCRI score. Only 16% of the population had insulin requiring diabetes mellitus (Table 1).

**Table 1:** Baseline characteristics - elective and emergency surgeries

**Elective surgery**					
	**Group I**	**Group II (score = 1)**	**Group III (score = 2)**	**Group IV (score ≥ 3)**	**Overall**
Total	0	768 (15)	2900 (57)	1426 (28)	5094 (28)
**Demographics**					
Age, [IQR], y	-	54.7 [45.2–62.8]	60.3 [49.1–70.1]	64.2 [54.6–71.8]	60.4 [50.0–69.7]
Male gender (%)	-	458 (60)	1912 (66)	955 (67)	3325 (65)
**Dialysis modality**					
Haemodialysis (%)	-	0	1397 (48)	841 (59)	2238 (44)
Peritoneal dialysis (%)	-	0	1029 (35)	450 (32)	1479 (29)
Home haemodialysis (%)	-	0	173 (6)	51 (4)	224 (4)
Kidney transplant (%)	-	768 (100)	301 (10)	84 (6)	1153 (23)
**Comorbidities**					
Ischaemic heart disease (%)	-	0	63 (2)	745 (52)	808 (16)
Diabetes mellitus requiring insulin (%)	-	0	58 (2)	621 (44)	679 (13)
Cerebrovascular disease (%)	-	0	17 (1)	141 (10)	158 (3)
Heart failure/cardiomyopathy (%)	-	0	32 (1)	450 (32)	482 (10)
MACE		7 (1)	59 (2)	87 (6)	153 (3)
**Emergency surgery**					
	**Group I (score = 0)**	**Group II (score = 1)**	**Group III (score = 2)**	**Group IV (score ≥ 3)**	**Overall**
Total	0	360 (11)	1688 (51)	1259 (38)	3307
**Demographics**					
Age, [IQR], year	-	52.4 [40.9–61.1]	59.5 [47.0–70.9]	63.6 [53.5–72.3]	60.2 [48.7–70.6]
Male gender (%)	-	198 (55)	956 (57)	775 (62)	1929 (58)
**Dialysis modality**					
Haemodialysis (%)	-	-	773 (46)	703 (56)	1476 (45)
Peritoneal dialysis (%)	-	-	588 (35)	445 (35)	1033 (31)
Home haemodialysis (%)	-	-	97 (6)	46 (4)	143 (4)
Kidney transplant (%)	-	360 (100)	230 (14)	65 (5)	655 (20)
**Comorbidities**					
Ischaemic heart disease (%)	-	0	42 (3)	670 (53)	712 (22)
Diabetes mellitus requiring insulin (%)	-	0	76 (5)	469 (37)	545 (16)
Cerebrovascular disease (%)	-	0	9 (1)	157 (12)	166 (5)
Heart failure/cardiomyopathy (%)	-	0	29 (2)	515 (41)	544 (16)
MACE	-	29 (8.1)	231 (13.7)	317 (25.2)	577 (17.4)

MACE occurred in 153 individuals (3.0%). Rates increased with higher RCRI scores. Odds ratios from multivariable regression for individual variables are shown in Table 2. Multivariable logistic regression showed that a history of ischaemic heart disease had the largest odds ratio, followed by heart failure. When the analyses were repeated stratified by age group and by KRT modality, both ischaemic heart disease and heart failure remained significant.

**Table 2:** Odds ratio of individual variables from multivariable regression

		**All elective surgeries by subgroup**				
**Variable**	**All elective surgeries (95% ** ***CI*** **)**	** < 65 years (95% ** ***CI*** **)**	** ≥ 65 years (95% ** ***CI*** **)**	**Dialysis only (95% ** ***CI*** **)**	**Transplant only (95% ** ***CI*** **)**	**All emergency (95% ** ***CI*** **)**
Ischaemic heart disease	3.02 (2.11–4.33)	4.33 (2.45–7.66)	2.03 (1.28–3.23	3.31 (2.25–4.87)	1.92 (0.72–5.13)	1.94 (1.60–2.42)
Diabetes mellitus requiring insulin	1.00 (0.65–1.57)	1.25 (0.66–2.34)	0.92 (0.49–1.74)	1.00 (0.63–1.61)	1.14 (0.31–4.19)	0.96 (0.75–1.23)
Cerebrovascular disease	0.77 (0.33–1.81)	1.06 (0.31–3.61)	0.56 (0.17–1.84)	0.90 (0.38–2.11)	-	1.64 (1.15–2.36)
Heart failure/cardiomyopathy	2.83 (1.91–4.18)	2.84 (1.54–5.23)	2.75 (1.65–4.58)	2.23 (1.45–3.45)	9.27 (3.77–22.80)	1.73 (1.38–2.17)
High risk surgery		-	-	-	-	-
Kidney failure	1.49 (0.90–2.48)	2.08 (0.94–4.64)	0.74 (0.38–1.43)	-	2.05 (0.81–5.19)	1.62 (1.21–2.16)

**Fig. 2:**
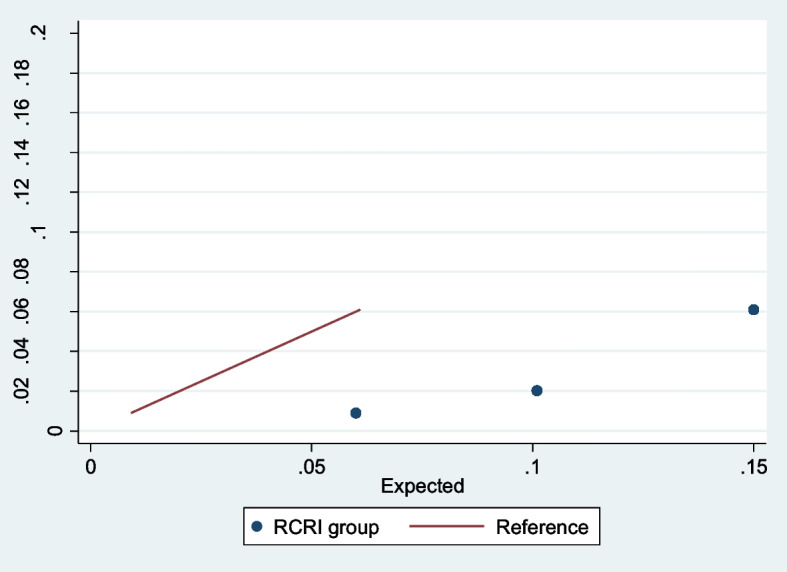
Calibration plot (all elective surgery). Observed risk of MACE plotted against the predicted. Solid line represented perfect calibration. Solid dots represent grouping of predicted risks. Grouped estimates are below the dashed line suggestive over overestimation of risk

Overall, RCRI had poor discrimination in patients on chronic KRT undergoing elective surgery (*AUROC* 0.67), particularly in patients aged greater than 65 years (*AUROC* 0.59, Table 3). Calibration plot showed that RCRI overestimated risk for MACE. The expected-to-observed outcome ratios were 6.0, 5.1 and 2.5 for those with RCRI scores of 1, 2 and ≥ 3, respectively (Fig. 2).

**Table 3:** Results of discrimination analysis according by age group and KRT modality

Patient cohort	AUROC (95% *CI*)
All elective surgery (*n* = 5094)	0.67 (0.63–0.70)
All elective surgery age < 65 years (*n* = 3141)	0.74 (0.69–0.79)
All elective surgery age > 65 years (*n* = 1953)	0.59 (0.54–0.65)
All elective surgery in dialysis patients only (*n* = 3941)	0.65 (0.60–0.69)
All elective surgery in transplant patients only (*n* = 1153)	0.72 (0.62–0.82)
All emergency surgery (*n* = 3307)	0.61 (0.59–0.64)

Discrimination was moderate in patients under 65 years and in kidney transplant recipients, with AUROC values of 0.74 and 0.72, respectively. In patients under 65 years, the expected-to-observed ratios were 0.7, 5.1 and 2.5, and for kidney transplant recipients, the ratios were 0.7, 2.7 and 1.8 for RCRI scores of 1, 2 and ≥ 3, respectively (Supplementary Fig. 1A, B, C, D, E). Decision curve analysis showed that there was no net benefit of using the RCRI tool in determining the use of HDU and intensive monitoring postoperatively in patients under 65 years, but a slight benefit associated with threshold probability > 5.5% in kidney transplant recipients (Supplementary Fig. 2A, B, C, D, E).

**Fig. 3:**
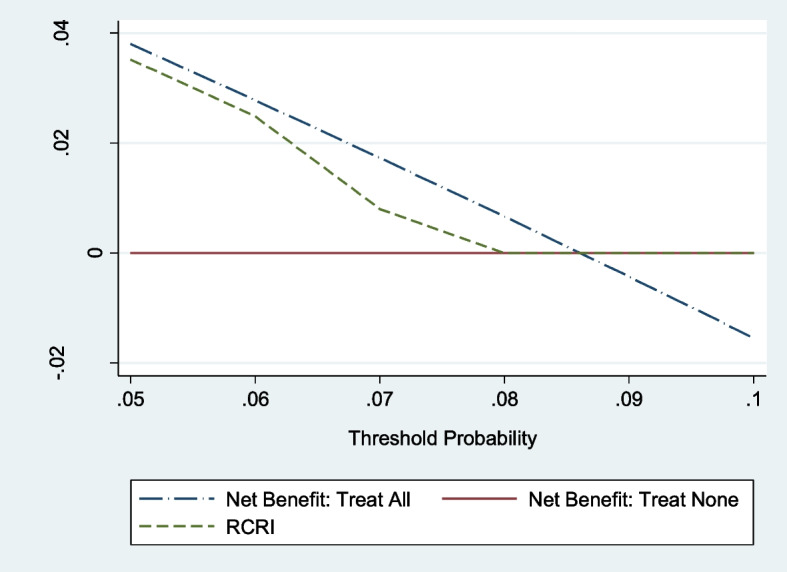
Decision curve analysis (all elective surgeries) plotting net benefit against threshold probability, comparing the clinical usefulness of RCRI versus strategies of all patients having surgery (green dashed line) and no participants having surgery (solid red line)

In exploratory analysis of 3307 patients undergoing emergency abdominal surgery, MACE occurred in 577 patients (17.4%). More than a quarter (25.2%) of the patients who had an RCRI score of 3 or more experienced a MACE. All comorbidities, with the exception of insulin-requiring diabetes mellitus, were significantly associated with MACE in the multivariable analysis (Table 2). However, the model had poor discrimination with AUROC of 0.613. Here, the model underestimated the risk, with the expected-to-observed ratios being 0.8, 0.7 and 0.5 for those with RCRI scores of 1, 2 or ≥ 3, respectively (Supplementary Fig. 1A, B, C, D, E). Decision curve analysis also showed limited benefit and in fact showed potential harm at a threshold probability of > 9.5% (Fig. 3).

## Discussion

This external validation study evaluated the performance of RCRI in Australian and New Zealand patients receiving chronic KRT and undergoing elective abdominal surgery between 2000 and 2015. This study found that the RCRI performed poorly in this patient group, and clinicians needed to be cautious in its interpretation preoperatively.

Our analysis showed poor discrimination in predicting MACE from RCRI (*AUROC* 0.67), and overestimation of risk in all RCRI categories, particularly in patients older than 65 and dialysis patients. Patients on chronic dialysis undergoing abdominal surgery automatically scored 2 points because of the severity of kidney failure and abdominal surgery being classified as high-risk surgery. Therefore, most of the patients receiving dialysis (HD, HHD and PD) were stratified only into two categories: score of 2 or score ≥ 3, which may explain the model’s poor discriminatory ability.

Overestimation of risk was especially the case in those with a score of 2 or more which made up 84.9% of the validation cohort. Although the magnitude of overestimation decreased with a higher RCRI score (≥ 3), it remained more than two and half times that predicted, suggesting that that the nuances of dialysis therapies such as dialysis vintage and modality need further consideration in risk assessment (Palamuthusingam et al. 2022). Although the model performed better when evaluating kidney transplant patients only, overestimation of risk remained a concern. Decision curve analysis in kidney transplant patients showed that if an individual’s threshold risk was 5%, the use of the estimated RCRI risk was of benefit in terms of deciding about utilising HDU monitoring and proactive cardiac marker testing (3 in 100 patients).

These findings were consistent with a prior North American study which evaluated the use of RCRI in 9917 patients with kidney failure undergoing various surgeries. This study also demonstrated poor model performance (*AUROC* 0.64 95% *CI* 0.62–0.65) and overestimation of postoperative MACE risk (Harrison et al. 2022). Our study extends current understanding by demonstrating that the utility of the RCRI is different depending on KRT modality and age. In addition, to minimise the heterogeneity of surgical procedures and the variable operative risk associated with different types of surgery (e.g. certain vascular surgeries are higher risk than abdominal surgery), our study also showed that even when examining surgeries of moderate risk (abdominal) only, the RCRI did not perform well.

RCRI remains one of the most used and validated risk assessment tools, despite being derived from a single centre using just over 4315 patients more than two decades ago (Lee et al. 1999). Differences in outcome definition and ascertainment may in part explain the model’s observed poor performance in patients receiving chronic KRT. The original derivation study’s definition of myocardial infarction utilised a combination of biochemical and electrocardiographic changes, with data extraction performed by study personnel. On the other hand, this validation study used ICD-10-AM codes obtained by clinical coders. Having said that, appropriate assignment of ICD code is contingent on the clarity of medical documentation (Palamuthusingam, et al. 2022). Importantly, only 4% of the 2893 original derivation cohort had a serum creatinine > 177 µmol/L, with the proportion of patients on dialysis not disclosed. Therefore, it is arguable that this model should not be used in patients with advanced kidney failure in the first place (Lee et al. 1999).

Exploratory analysis of emergency surgeries only showed poor discrimination of the RCRI model. However, in these circumstances, RCRI underestimated risk and in fact may have led to harm if the risk was estimated at greater than 9.5%. These findings are not surprising, given that the original derivation study did not include emergency surgery, and its use in the general population is also discouraged. There are other existing risk assessment tools, such as POSSUM, ACS-NSQIP, SORT and SRS, which are also commonly used and, like the RCRI, were not derived nor have been validated in patients on chronic KRT (Palamuthusingam et al. 2019). The ASA-PS classification system is frequently used to measure a patient’s physiological reserve based on their systemic illness, although it was not designed as a predictive tool for perioperative risk. As such, patients on chronic dialysis are consistently classified as physical class III due to their reliance on dialysis, placing them in the second-highest risk category and neglecting their actual functional ability. Furthermore, the subjective nature of the ASA-PS results in low inter-rater reliability, leading to an overreliance on comorbidity assessments (Riley et al. 2014).

The strength of this study is the large number of consecutive patients undergoing abdominal surgery across all jurisdictions in Australia and New Zealand, increasing the generalisability of the findings. However, there are a number of limitations to be considered. Firstly, due to the retrospective nature of the study, indication bias is possible as there may have been patients who required surgery but did not proceed to theatre due to the presence of multiple comorbidities and therefore potentially contributing to the tool’s overestimation of risk. Secondly, coding bias was possible as the study relied on the use of administrative datasets to ascertain outcomes that were not able to be validated by biochemical findings, such as an elevated troponin to confirm a myocardial infarction. However, in Australia, there are comprehensive coding standards (Australian Coding Standards) with documented data definitions. In addition, the skills and education of clinical coders, the degree of professional coding supervision, and the existence and rigour of regular coding audits to identify systematic errors in their records all strengthened the validity and robustness of routinely collected hospital data used in this analysis (Palamuthusingam, et al. 2020, 2022; Elsworthy and S.M.C., B. Graham, Y. Guo, K. C. Innes, C. L. Loggie, N. M. Rankin, P. M. Saad, I. H. Soo L. M. Tun 2013). Thirdly, the reference values used to assess calibration were obtained from the original derivation study. As such, variation in surgical practice patterns and healthcare systems between regions can impact the tool’s performance. Fourthly, as no patient on chronic KRT will be in Group I because they all have an elevated serum creatinine, patients were categorised into three unevenly sized categories (with a majority in Group II), reducing the discriminatory value of the tool. Finally, the generalisability of the findings to other surgery types is limited.

## Conclusion

RCRI remains an easy-to-use bedside risk assessment tool for many clinicians. However, recommendations regarding its use in patients on chronic KRT for risk stratification need to be tempered due to its poor performance. Future studies need to incorporate dialysis and kidney transplant-specific characteristics to improve risk assessment and inform decision-making.

### Supplementary Information

Supplementary Material 1. Supplementary figures: Fig. 1A-E: Calibration plots: Observed risk of MACE plotted against the predicted. Solid line represented perfect calibration. Solid dots represent grouping of predicted risks. Grouped estimates are below the dashed line suggestive over overestimation of risk. A: Calibration plot (all elective surgery < 65). B: Calibration plot (all elective surgery ≥ 65). C: Calibration plot (all elective surgery in transplant patient only). D: Calibration plot (all elective surgery in dialysis patient only). E: Calibration plot (all emergency surgeries only). Supplementary Fig. 2: Decision curve analysis (all elective surgeries) plotting net benefit against threshold probability, comparing the clinical usefulness of RCRI versus strategies of all patients having surgery (green dashed line) and no participants having surgery (solid red line). A: Decision curve analysis (all elective surgeries < 65). B: Decision curve analysis (all elective surgeries ≥ 65). C: Decision curve analysis (all elective surgeries in transplant patients only). D: Decision curve analysis (all elective surgeries in transplant patients only). E: Decision curve analysis (all emergency surgeries). Supplementary tables: Supplementary Table 1: ICD-10AM codes for comorbidities. Supplementary Table 2: ACHI codes for abdominal surgery. Supplementary Table 3: ICD-10AM codes for MACE events. Supplementary Table 4: Types of surgery (Elective vs. Emergency procedures).

## Data Availability

No datasets were generated or analysed during the current study.
