# Supplementary material for: Revised cardiac risk index in predicting cardiovascular complications in patients receiving chronic kidney replacement therapy undergoing elective general surgery
Source: Perioper Med (Lond). 2024 Jul 10;13:70. doi: 10.1186/s13741-024-00429-0 (PMC11234675; doi:10.1186/s13741-024-00429-0)
Supplement: Supplementary file 1 — Supplementary Material 1. Supplementary figures: Fig. 1A-E: Calibration plots: Observed risk of MACE plotted against the predicted. Solid line represented perfect calibration. Solid dots represent grouping of predicted risks. Grouped estimates are below the dashed line suggestive over overestimation of risk. A: Calibration plot (all elective surgery < 65). B: Calibration plot (all elective surgery ≥ 65). C: Calibration plot (all elective surgery in transplant patient only). D: Calibration plot (all elective surgery in dialysis patient only). E: Calibration plot (all emergency surgeries only). Supplementary Fig. 2: Decision curve analysis (all elective surgeries) plotting net benefit against threshold probability, comparing the clinical usefulness of RCRI versus strategies of all patients having surgery (green dashed line) and no participants having surgery (solid red line). A: Decision curve analysis (all elective surgeries < 65). B: Decision curve analysis (all elective surgeries ≥ 65). C: Decision curve analysis (all elective surgeries in transplant patients only). D: Decision curve analysis (all elective surgeries in transplant patients only). E: Decision curve analysis (all emergency surgeries). Supplementary tables: Supplementary Table 1: ICD-10AM codes for comorbidities. Supplementary Table 2: ACHI codes for abdominal surgery. Supplementary Table 3: ICD-10AM codes for MACE events. Supplementary Table 4: Types of surgery (Elective vs. Emergency procedures). [file 13741_2024_429_MOESM1_ESM.docx]

**Supplementary Table 1:** ICD-10AM codes for comorbidities

**Diabetes Mellitus:**

"E090" Impaired glucose regulation with peripheral angiopathy

"E0901" Impaired glucose regulation with peripheral angiopathy without gangrene

"E0902" Impaired glucose regulation with peripheral angiopathy with gangrene

"E091" Impaired glucose regulation with features of insulin resistance

"E092" Intermediate hyperglycaemia with kidney complication

"E0921" Intermediate hyperglycaemia with incipient nephropathy

"E0929" Intermediate hyperglycaemia with other specified kidney complication

"E093" Intermediate hyperglycaemia with ophthalmic complication

"E0931" Intermediate hyperglycaemia with background retinopathy

"E0932" Intermediate hyperglycaemia with preproliferative retinopathy

"E094" Intermediate hyperglycaemia with neurological complication

"E0940" Intermediate hyperglycaemia with unspecified neuropathy

"E0942" Intermediate hyperglycaemia with polyneuropathy

"E095" Intermediate hyperglycaemia with peripheral angiopathy

"E0951" Intermediate hyperglycaemia with peripheral angiopathy, without gangrene

"E0952" Intermediate hyperglycaemia with peripheral angiopathy, with gangrene

"E097" Intermediate hyperglycaemia with multiple complications

"E0971" Intermediate hyperglycaemia with multiple microvascular complications

"E0972" Intermediate hyperglycaemia with features of insulin resistance

"E13" Other specified diabetes mellitus

"E097" Intermediate hyperglycaemia with multiple complications

"E098" Intermediate hyperglycaemia with unspecified complication

"E099" Intermediate hyperglycaemia without complication

"E10" Type 1 diabetes mellitus

"E100" Type 1 diabetes mellitus with hyperosmolarity

"E1000" Insulin-dependent diabetes mellitus with coma, not stated as uncontrolled

"E1001" Type 1 diabetes mellitus with hyperosmolarity without nonketotic hyperglycaemic-hyperosmolar coma (NKHHC)

"E1002" Type 1 diabetes mellitus with hyperosmolarity with coma

"E101" Type 1 diabetes mellitus with acidosis

"E1010" Insulin-dependent diabetes mellitus with ketoacidosis, not stated as uncontrolled

"E1011" Type 1 diabetes mellitus with ketoacidosis, without coma

"E1012" Type 1 diabetes mellitus with ketoacidosis, with coma

"E1013" Type 1 diabetes mellitus with lactic acidosis, without coma

"E1014" Type 1 diabetes mellitus with lactic acidosis, with coma

"E1015" Type 1 diabetes mellitus with ketoacidosis, with lactic acidosis, without coma

"E1016" Type 1 diabetes mellitus with ketoacidosis, with lactic acidosis, with coma

"E102" Type 1 diabetes mellitus with kidney complication

"E1020" Type 1 diabetes mellitus with renal complication, unspecified

"E1021" Type 1 diabetes mellitus with incipient diabetic nephropathy

"E1022" Type 1 diabetes mellitus with established diabetic nephropathy

"E1023" Type 1 diabetes mellitus with advanced renal disease

"E1029" Type 1 diabetes mellitus with other specified kidney complication

"E103" Type 1 diabetes mellitus with ophthalmic complication

"E1030" Type 1 diabetes mellitus with ophthalmic complication, unspecified

"E1031" Type 1 diabetes mellitus with background retinopathy

"E1032" Type 1 diabetes mellitus with preproliferative retinopathy

"E1033" Type 1 diabetes mellitus with proliferative retinopathy

"E1034" Type 1 diabetes mellitus with other retinopathy

"E1035" Type 1 diabetes mellitus with advanced ophthalmic disease

"E1036" Type 1 diabetes mellitus with diabetic cataract

"E1039" Type 1 diabetes mellitus with other specified ophthalmic complication

"E104" Type 1 diabetes mellitus with neurological complication

"E1040" Type 1 diabetes mellitus with unspecified neuropathy

"E1041" Type 1 diabetes mellitus with diabetic mononeuropathy

"E1042" Type 1 diabetes mellitus with diabetic polyneuropathy

"E1043" Type 1 diabetes mellitus with diabetic autonomic neuropathy

"E1049" Type 1 diabetes mellitus with other specified neurological complication

"E105" Type 1 diabetes mellitus with circulatory complication

"E1050" Type 1 diabetes mellitus with circulatory complication, unspecified

"E1051" Type 1 diabetes mellitus with peripheral angiopathy, without gangrene

"E1052" Type 1 diabetes mellitus with peripheral angiopathy, with gangrene

"E1053" Type 1 diabetes mellitus with diabetic cardiomyopathy

"E1059" Type 1 diabetes mellitus with other specified circulatory complication

"E106" Type 1 diabetes mellitus with other specified complication

"E1060" Insulin-dependent diabetes mellitus with other specified complications, not stated as uncontrolled

"E1061" Type 1 diabetes mellitus with specified diabetic musculoskeletal and connective tissue complication

"E1062" Type 1 diabetes mellitus with specified skin and subcutaneous tissue complication

"E1063" Type 1 diabetes mellitus with specified periodontal complication

"E1064" Type 1 diabetes mellitus with hypoglycaemia

"E1065" Type 1 diabetes mellitus with poor control

"E1069" Type 1 diabetes mellitus with other specified complication

"E107" Type 1 diabetes mellitus with multiple complications

"E1070" Insulin-dependent diabetes mellitus with multiple complications, not stated as uncontrolled

"E1071" Type 1 diabetes mellitus with multiple microvascular and other specified nonvascular complications

"E1073" Type 1 diabetes mellitus with foot ulcer due to multiple causes

"E108" Type 1 diabetes mellitus with unspecified complication

"E1080" Insulin-dependent diabetes mellitus with unspecified complications, not stated as uncontrolled

"E1081" Insulin-dependent diabetes mellitus with unspecified complications, stated as uncontrolled

"E109" Type 1 diabetes mellitus without complication

"E1090" Insulin-dependent diabetes mellitus without complications, not stated as uncontrolled

"E1091" Insulin-dependent diabetes mellitus without complications, stated as uncontrolled

"E11" Type 2 diabetes mellitus

"E110" Type 2 diabetes mellitus with hyperosmolarity

"E1100" Non-insulin-dependent diabetes mellitus with coma, not stated as uncontrolled

"E1101" Type 2 diabetes mellitus with hyperosmolarity without nonketotic hyperglycaemic-hyperosmolar coma (NKHHC)

"E1102" Type 2 diabetes mellitus with hyperosmolarity with coma

"E111" Type 2 diabetes mellitus with acidosis

"E1110" Non-insulin-dependent diabetes mellitus with ketoacidosis, not stated as uncontrolled

"E1111" Type 2 diabetes mellitus with ketoacidosis, without coma

"E1112" Type 2 diabetes mellitus with ketoacidosis, with coma

"E1113" Type 2 diabetes mellitus with lactic acidosis, without coma

"E1114" Type 2 diabetes mellitus with lactic acidosis, with coma

"E1115" Type 2 diabetes mellitus with ketoacidosis, with lactic acidosis, without coma

"E1116" Type 2 diabetes mellitus with ketoacidosis, with lactic acidosis, with coma

"E112" Type 2 diabetes mellitus with kidney complication

"E1120" Type 2 diabetes mellitus with renal complication, unspecified

"E1121" Type 2 diabetes mellitus with incipient diabetic nephropathy

"E1122" Type 2 diabetes mellitus with established diabetic nephropathy

"E1123" Type 2 diabetes mellitus with advanced renal disease

"E1129" Type 2 diabetes mellitus with other specified kidney complication

"E113" Type 2 diabetes mellitus with ophthalmic complication

"E1130" Type 2 diabetes mellitus with ophthalmic complication, unspecified

"E1131" Type 2 diabetes mellitus with background retinopathy

"E1132" Type 2 diabetes mellitus with preproliferative retinopathy

"E1133" Type 2 diabetes mellitus with proliferative retinopathy

"E1134" Type 2 diabetes mellitus with other retinopathy

"E1135" Type 2 diabetes mellitus with advanced ophthalmic disease

"E1136" Type 2 diabetes mellitus with diabetic cataract

"E1139" Type 2 diabetes mellitus with other specified ophthalmic complication

"E114" Type 2 diabetes mellitus with neurological complication

"E1140" Type 2 diabetes mellitus with unspecified neuropathy

"E1141" Type 2 diabetes mellitus with diabetic mononeuropathy

"E1142" Type 2 diabetes mellitus with diabetic polyneuropathy

"E1143" Type 2 diabetes mellitus with diabetic autonomic neuropathy

"E1149" Type 2 diabetes mellitus with other specified neurological complication

"E115" Type 2 diabetes mellitus with circulatory complication

"E1151" Type 2 diabetes mellitus with peripheral angiopathy, without gangrene

"E1152" Type 2 diabetes mellitus with peripheral angiopathy, with gangrene

"E1153" Type 2 diabetes mellitus with diabetic cardiomyopathy

"E1159" Type 2 diabetes mellitus with other specified circulatory complication

"E116" Type 2 diabetes mellitus with other specified complication

"E1160" Non-insulin-dependent diabetes mellitus with other specified complications, not stated as uncontrolled

"E1161" Type 2 diabetes mellitus with specified diabetic musculoskeletal and connective tissue complication

"E1162" Type 2 diabetes mellitus with specified skin and subcutaneous tissue complication

"E1169" Type 2 diabetes mellitus with other specified complication

"E117" Type 2 diabetes mellitus with multiple complications

"E1170" Non-insulin-dependent diabetes mellitus with multiple complications, not stated as uncontrolled

"E1171" Type 2 diabetes mellitus with multiple microvascular and other specified nonvascular complications

"E1172" Type 2 diabetes mellitus with features of insulin resistance

"E1173" Type 2 diabetes mellitus with foot ulcer due to multiple causes

"E118" Type 2 diabetes mellitus with unspecified complication

"E1180" Non-insulin-dependent diabetes mellitus with unspecified complications, not stated as uncontrolled

"E1181" Non-insulin-dependent diabetes mellitus with unspecified complications, stated as uncontrolled

"E119" Type 2 diabetes mellitus without complication

"E1190" Non-insulin-dependent diabetes mellitus without complications, not stated as uncontrolled

"E1191" Non-insulin-dependent diabetes mellitus without complications, stated as uncontrolled

"E13" Other specified diabetes mellitus

"E130" Other specified diabetes mellitus with hyperosmolarity

"E1300" Other specified diabetes mellitus with coma, not stated as uncontrolled

"E1301" Other specified diabetes mellitus with hyperosmolarity, without nonketotic hyperglycaemic-hyperosmolar coma (NKHHC)

"E1302" Other specified diabetes mellitus with hyperosmolarity, with coma

"E131" Other specified diabetes mellitus with acidosis

"E1310" Other specified diabetes mellitus with ketoacidosis, not stated as uncontrolled

"E1311" Other specified diabetes mellitus with ketoacidosis, without coma

"E1312" Other specified diabetes mellitus with ketoacidosis, with coma

"E1313" Other specified diabetes mellitus with lactic acidosis, without coma

"E1314" Other specified diabetes mellitus with lactic acidosis, with coma

"E1315" Other specified diabetes mellitus with ketoacidosis, with lactic acidosis, without coma

"E1316" Other specified diabetes mellitus with ketoacidosis, with lactic acidosis, with coma

"E132" Other specified diabetes mellitus with kidney complication

"E1320" Other specified diabetes mellitus with renal complication, unspecified

"E1321" Other specified diabetes mellitus with incipient diabetic nephropathy

"E1322" Other specified diabetes mellitus with established diabetic nephropathy

"E1323" Other specified diabetes mellitus with advanced renal disease

"E1329" Other specified diabetes mellitus with other specified kidney complication

"E133" Other specified diabetes mellitus with ophthalmic complication

"E1330" Other specified diabetes mellitus with ophthalmic complication, unspecified

"E1331" Other specified diabetes mellitus with background retinopathy

"E1332" Other specified diabetes mellitus with preproliferative retinopathy

"E1333" Other specified diabetes mellitus with proliferative retinopathy

"E1334" Other specified diabetes mellitus with other retinopathy

"E1335" Other specified diabetes mellitus with advanced ophthalmic disease

"E1336" Other specified diabetes mellitus with diabetic cataract

"E1339" Other specified diabetes mellitus with other specified ophthalmic complication

"E134" Other specified diabetes mellitus with neurological complication

"E1340" Other specified diabetes mellitus with unspecified neuropathy

"E1341" Other specified diabetes mellitus with diabetic mononeuropathy

"E1342" Other specified diabetes mellitus with diabetic polyneuropathy

"E1343" Other specified diabetes mellitus with diabetic autonomic neuropathy

"E1349" Other specified diabetes mellitus with other specified neurological complication

"E135" Other specified diabetes mellitus with circulatory complication

"E1350" Other specified diabetes mellitus with circulatory complication, unspecified

"E1351" Other specified diabetes mellitus with peripheral angiopathy, without gangrene

"E1352" Other specified diabetes mellitus with peripheral angiopathy, with gangrene

"E1353" Other specified diabetes mellitus with diabetic cardiomyopathy

"E1359" Other specified diabetes mellitus with other specified circulatory complication

"E136" Other specified diabetes mellitus with other specified complication

"E1360" Other specified diabetes mellitus with other specified complications, not stated as uncontrolled

"E1361" Other specified diabetes mellitus with specified diabetic musculoskeletal and connective tissue complication

"E1362" Other specified diabetes mellitus with specified skin and subcutaneous tissue complication

"E1363" Other specified diabetes mellitus with specified periodontal complication

"E1364" Other specified diabetes mellitus with hypoglycaemia

"E1365" Other specified diabetes mellitus with poor control

"E1369" Other specified diabetes mellitus with other specified complication

"E137" Other specified diabetes mellitus with multiple complications

"E1370" Other specified diabetes mellitus with multiple complications, not stated as uncontrolled

"E1371" Other specified diabetes mellitus with multiple microvascular and other specified nonvascular complications

"E1372" Other specified diabetes mellitus with features of insulin resistance

"E1373" Other specified diabetes mellitus with foot ulcer due to multiple causes

"E138" Other specified diabetes mellitus with unspecified complication

"E1380" Other specified diabetes mellitus with unspecified complications, not stated as uncontrolled

"E1381" Other specified diabetes mellitus with unspecified complications, stated as uncontrolled

"E139" Other specified diabetes mellitus without complication

"E1390" Other specified diabetes mellitus without complications, not stated as uncontrolled

"E1391" Other specified diabetes mellitus without complications, stated as uncontrolled

"E14" Unspecified diabetes mellitus

"E140" Unspecified diabetes mellitus with hyperosmolarity

"E1400" Unspecified diabetes mellitus with coma, not stated as uncontrolled

"E1401" Unspecified diabetes mellitus with hyperosmolarity, without nonketotic hyperglycaemic-hyperosmolar coma (NKHHC)

"E1402" Unspecified diabetes mellitus with hyperosmolarity, with coma

"E141" Unspecified diabetes mellitus with acidosis

"E1410" Unspecified diabetes mellitus with ketoacidosis, not stated as uncontrolled

"E1411" Unspecified diabetes mellitus with ketoacidosis, without coma

"E1412" Unspecified diabetes mellitus with ketoacidosis, with coma

"E1413" Unspecified diabetes mellitus with lactic acidosis, without coma

"E1414" Unspecified diabetes mellitus with lactic acidosis, with coma

"E1415" Unspecified diabetes mellitus with ketoacidosis, with lactic acidosis, without coma

"E1416" Unspecified diabetes mellitus with ketoacidosis, with lactic acidosis, with coma

"E142" Unspecified diabetes mellitus with kidney complication

"E1420" Unspecified diabetes mellitus with renal complication, unspecified

"E1421" Unspecified diabetes mellitus with incipient diabetic nephropathy

"E1422" Unspecified diabetes mellitus with established diabetic nephropathy

"E1423" Unspecified diabetes mellitus with advanced renal disease

"E1429" Unspecified diabetes mellitus with other specified kidney complication

"E143" Unspecified diabetes mellitus with ophthalmic complication

"E1430" Unspecified diabetes mellitus with ophthalmic complication, unspecified

"E1431" Unspecified diabetes mellitus with background retinopathy

"E1432" Unspecified diabetes mellitus with preproliferative retinopathy

"E1433" Unspecified diabetes mellitus with proliferative retinopathy

"E1434" Unspecified diabetes mellitus with other retinopathy

"E1435" Unspecified diabetes mellitus with advanced ophthalmic disease

"E1436" Unspecified diabetes mellitus with diabetic cataract

"E1439" Unspecified diabetes mellitus with other specified ophthalmic complication

"E144" Unspecified diabetes mellitus with neurological complication

"E1440" Unspecified diabetes mellitus with unspecified neuropathy

"E1441" Unspecified diabetes mellitus with diabetic mononeuropathy

"E1442" Unspecified diabetes mellitus with diabetic polyneuropathy

"E1443" Unspecified diabetes mellitus with diabetic autonomic neuropathy

"E1449" Unspecified diabetes mellitus with other specified neurological complication

"E145" Unspecified diabetes mellitus with circulatory complication

"E1450" Unspecified diabetes mellitus with peripheral circulatory complications, not stated as uncontrolled

"E1451" Unspecified diabetes mellitus with peripheral angiopathy, without gangrene

"E1452" Unspecified diabetes mellitus with peripheral angiopathy, with gangrene

"E1453" Unspecified diabetes mellitus with diabetic cardiomyopathy

"E1459" Unspecified diabetes mellitus with other specified circulatory complication

"E146" Unspecified diabetes mellitus with other specified complication

"E1460" Unspecified diabetes mellitus with other specified complications, not stated as uncontrolled

"E1461" Unspecified diabetes mellitus with specified diabetic musculoskeletal and connective tissue complication

"E1462" Unspecified diabetes mellitus with specified skin and subcutaneous tissue complication

"E1463" Unspecified diabetes mellitus with specified periodontal complication

"E1464" Unspecified diabetes mellitus with hypoglycaemia

"E1465" Unspecified diabetes mellitus with poor control

"E1469" Unspecified diabetes mellitus with other specified complication

"E147" Unspecified diabetes mellitus with multiple complications

"E1470" Unspecified diabetes mellitus with multiple complications, not stated as uncontrolled

"E1471" Unspecified diabetes mellitus with multiple microvascular and other specified nonvascular complications

"E1471" Unspecified diabetes mellitus with multiple microvascular and other specified nonvascular complications

"E1472" Unspecified diabetes mellitus with features of insulin resistance

"E1473" Unspecified diabetes mellitus with foot ulcer due to multiple causes

"E148" Unspecified diabetes mellitus with unspecified complication

"E1480" Unspecified diabetes mellitus with unspecified complications, not states as uncontrolled

"E1481" Unspecified diabetes mellitus with unspecified complications, stated as uncontrolled

"E149" Unspecified diabetes mellitus without complication

"E1490" Unspecified diabetes mellitus without complications, not stated as uncontrolled

"E1491" Unspecified diabetes mellitus without complications, stated

**ISCHAEMIC HEART DISEASE:**

"I210" Acute transmural myocardial infarction of anterior wall

"I21" Acute myocardial infarction

"I211" Acute transmural myocardial infarction of inferior wall

"I212" Acute transmural myocardial infarction of other sites

"I213" Acute transmural myocardial infarction of unspecified site

"I214" Acute subendocardial myocardial infarction

"I219" Acute myocardial infarction, unspecified

"I22" Subsequent myocardial infarction

"I220" Subsequent myocardial infarction of anterior wall

"I221" Subsequent myocardial infarction of inferior wall

"I228" Subsequent myocardial infarction of other sites

"I229" Subsequent myocardial infarction of unspecified site

"I23" Certain current complications following acute myocardial infarction

"I230" Haemopericardium as current complication following acute myocardial infarction

"I231" Atrial septal defect as current complication following acute myocardial infarction

"I232" Ventricular septal defect as current complication following acute myocardial infarction

"I233" Rupture of cardiac wall without haemopericardium as current complication following acute myocardial infarction

"I234" Rupture of chordae tendineae as current complication following acute myocardial infarction

"I235" Rupture of papillary muscle as current complication following acute myocardial infarction

"I236" Thrombosis of atrium, auricular appendage, and ventricle as current complications following acute myocardial infarction

"I238" Other current complications following acute myocardial infarction

"I24" Other acute ischaemic heart diseases

"I240" Coronary thrombosis not resulting in myocardial infarction

"I241" Dressler's syndrome

"I248" Other forms of acute ischaemic heart disease

"I249" Acute ischaemic heart disease, unspecified

"I200" Unstable angina

"I201" Angina pectoris with documented spasm

"I208" Other forms of angina pectoris

"I209" Angina pectoris, unspecified

"I250" Atherosclerotic cardiovascular disease, so described

"I251" Atherosclerotic heart disease

"I252" Old myocardial infarction

"I253" Aneurysm of heart

"I254" Coronary artery aneurysm and dissection

"I255" Ischaemic cardiomyopathy

"I256" Silent myocardial ischaemia

"I258" Other forms of chronic ischaemic heart disease

"I259" Chronic ischaemic heart disease, unspecified

"I2511" Atherosclerotic heart disease, of native coronary artery

"I2512" Atherosclerotic heart disease, of autologous bypass graft

"I2513" Atherosclerotic heart disease, of nonautologous bypass graft

"U821" Ischaemic heart disease

**CEREBROVASCULAR DISEASE**

“I672” Cerebral atherosclerosis

“I678” Other specified cerebrovascular diseases

“I679” Cerebrovascular disease, unspecified

“G459” Transient cerebral ischaemic attack, unspecified

“G453”Amaurosis fugax

"I693" Sequelae of cerebral infarction

“I610” Intracerebral haemorrhage in hemisphere, subcortical

“I611 Intracerebral haemorrhage in hemisphere, cortical

“I612” Intracerebral haemorrhage in hemisphere, unspecified

“I613” Intracerebral haemorrhage in brain stem

“I614” Intracerebral haemorrhage in cerebellum

“I615 Intracerebral haemorrhage, intraventricular

“I616” Intracerebral haemorrhage, multiple localised

“I618 Other intracerebral haemorrhage

“I619” Intracerebral haemorrhage, unspecified

“I61” Intracerebral haemorrhage

“I691” Sequelae of intracerebral haemorrhage

“S0623” Multiple intracerebral and cerebellar haematomas

“I630” Cerebral infarction due to thrombosis of precerebral arteries

“I631” Cerebral infarction due to embolism of precerebral arteries

“I632” Cerebral infarction due to unspecified occlusion or stenosis of precerebral arteries

“I634” Cerebral infarction due to embolism of cerebral arteries

“I635” Cerebral infarction due to unspecified occlusion or stenosis of cerebral arteries

“I636” Cerebral infarction due to cerebral venous thrombosis, nonpyogenic

“I638” Other cerebral infarction

“I639” Cerebral infarction, unspecified

“I63” Cerebral infarction

“G463” Brain stem stroke syndrome (I60-I67+)

“I64” Stroke, not specified as haemorrhage or infarction

“I694” Sequelae of stroke, not specified as haemorrhage or infarction

“I693” Sequelae of cerebral infarction

“I679” Cerebrovascular disease, unspecified

**CHORNIC OBSTRCUTIVE AIRWAY DISEASE**

"J440" Chronic obstructive pulmonary disease with acute lower respiratory infection

"J441" Chronic obstructive pulmonary disease with acute exacerbation, unspecified

"J448" Other specified chronic obstructive pulmonary disease

"J449" Chronic obstructive pulmonary disease, unspecified

"J43" Emphysema "J431" Panlobular emphysema

"J432" Centrilobular emphysema

"J438" Other emphysema

"J439" Emphysema, unspecified

"J44" Other chronic obstructive pulmonary disease

"U832" Chronic obstructive pulmonary disease

"U831" Emphysema, without mention of chronic obstructive pulmonary disease

"J410" Simple chronic bronchitis

"J411" Mucopurulent chronic bronchitis

"J47" Bronchiectasis

"U834" Bronchiectasis, without mention of cystic fibrosis

"J841" Other interstitial pulmonary diseases with fibrosis

"J848" Other specified interstitial pulmonary diseases

"J849" Interstitial pulmonary disease, unspecified

"I270" Primary pulmonary hypertension

"I272" Other secondary pulmonary hypertension

"I278" Other specified pulmonary heart diseases

"I28" Other diseases of pulmonary vessels

"I288" Other specified diseases of pulmonary vessels

"I289" Disease of pulmonary vessels, unspecified

"G473" Sleep apnoea "G4730" Sleep apnoea, unspecified

"G4731" Central sleep apnoea syndrome

"G4732" Obstructive sleep apnoea syndrome

"G4733" Sleep hypoventilation syndrome

"G4739" Other sleep apnoea

"I270" Primary pulmonary hypertension

"I272" Other secondary pulmonary hypertension

"I278" Other specified pulmonary heart diseases

"I28" Other diseases of pulmonary vessels

"I288" Other specified diseases of pulmonary vessels

"I289" Disease of pulmonary vessels, unspecified

"J841" Other interstitial pulmonary diseases with fibrosis

"J848" Other specified interstitial pulmonary diseases

"J849" Interstitial pulmonary disease, unspecified

**PERIPHERAL VASCULAR DISEASE**

"I700" Atherosclerosis of aorta

"I701" Atherosclerosis of renal artery

"I702" Atherosclerosis of arteries of extremities

"I708" Atherosclerosis of other arteries

"I709" Generalised and unspecified atherosclerosis

"I738" Other specified peripheral vascular diseases

"I739" Peripheral vascular disease, unspecified

"I73" Other peripheral vascular diseases

“I742" Embolism and thrombosis of arteries of upper extremities

“I743" Embolism and thrombosis of arteries of lower extremities

“I744" Embolism and thrombosis of arteries of extremities, unspecified

**HYPERTENSION**

"I10" Essential (primary) hypertension

“I11" Hypertensive heart disease

"I110" Hypertensive heart disease with (congestive) heart failure

"I119" Hypertensive heart disease without (congestive) heart failure

"I12" Hypertensive kidney disease

"I120" Hypertensive kidney disease with kidney failure

"I129" Hypertensive kidney disease without kidney failure

“I13" Hypertensive heart and kidney disease

"I130" Hypertensive heart and kidney disease with (congestive) heart failure

"I131" Hypertensive heart and kidney disease with kidney failure

"I132" Hypertensive heart and kidney disease with both (congestive) heart failure and kidney failure

"I139" Hypertensive heart and kidney disease, unspecified

"I15" Secondary hypertension

"I150" Renovascular hypertension

"I151" Hypertension secondary to other kidney disorders

"I152" Hypertension secondary to endocrine disorders

"I158" Other secondary hypertension

"I159" Secondary hypertension, unspecified

**HEART FAILURE/CARDIOMYOPATHY**

Supplementary Table 2: ACHI codes for abdominal surgery

**LARGE AND SMALL BOWEL SURGERY**

9030600 Laparoscopic insertion of feeding jejunostomy tube,

3056800 Endoscopic examination of small intestine via intraoperative enterotomy,

3037503 Enterotomy of small intestine,

3056900 Endoscopic examination of small intestine via laparotomy,

3056500 Resection of small intestine with formation of stoma,

3056600 Resection of small intestine with anastomosis,

3037509 Excision of Meckel's diverticulum,

3058000 Excision of lesion of duodenum,

3037501 Other enterostomy,

3037529 Temporary ileostomy,

3051501 Enterocolostomy,

3051502 Enteroenterostomy,

3206900 Formation of ileostomy reservoir,

4380700 Duodenoduodenostomy,

3037508 Reduction of intussusception of small intestine,

3037518 Reduction of volvulus of small intestine,

3056200 Closure of loop ileostomy,

3056201 Closure of ileostomy with restoration of bowel continuity, without resection, 3056204 Closure of other stoma of small intestine,

4381000 Repair of small intestine with single anastomosis,

4381001 Repair of small intestine with multiple anastomoses,

9030700 Other procedures on small intestine,

3218600 Intraoperative colonic lavage,

3037502 Colotomy,

3037523 Endoscopic examination of large intestine via laparotomy,

3051503 Ileocolic resection with anastomosis,

3051504 Laparoscopic ileocolic resection with anastomosis,

3051505 Ileocolic resection with formation of stoma,

3051506 Laparoscopic ileocolic resection with formation of stoma,

3200000 Limited excision of large intestine with formation of stoma,

3200001 Right hemicolectomy with formation of stoma,

3200002 Laparoscopic limited excision of large intestine with formation of stoma,

3200003 Laparoscopic right hemicolectomy with formation of stoma,

3200300 Limited excision of large intestine with anastomosis,

3200301 Right hemicolectomy with anastomosis, 3200303 Laparoscopic right hemicolectomy with anastomosis,

3200400 Sub-total colectomy with formation of stoma,

3200401 Extended right hemicolectomy with formation of stoma,

3200402 Laparoscopic subtotal colectomy with formation of stoma,

3200500 Subtotal colectomy with anastomosis,

3200501 Extended right hemicolectomy with anastomosis,

3200502 Laparoscopic subtotal colectomy with anastomosis,

3200503 Laparoscopic extended right hemicolectomy with anastomosis,

3200600 Left hemicolectomy with anastomosis,

3202900 Construction of colonic reservoir,

3037500 Caecostomy,

3037504 Other colostomy,

3037528 Temporary colostomy,

3037511 Reduction of intussusception of large intestine,

3037517 Reduction of volvulus of large intestine,

4380100 Correction of malrotation of intestine,

3037525 Suture of laceration of large intestine,

3038202 Radical repair of enterocutaneous fistula of large intestine,

3038203 Percutaneous repair of enterocutaneous fistula of large intestine,

3056202 Closure of loop colostomy,

3056203 Closure of colostomy with restoration of bowel continuity,

3056205 Closure of other stoma of large intestine,

3203300 Restoration of bowel continuity after Hartmann's procedure,

4381602 Other repair of large intestine,

9034001 Closure of fistula of large intestine,

9095100 Fixation of large intestine,

3056301 Revision of stoma of large intestine,

9031000 Other procedures on large intestine,

**ANORECTAL SURGERY**

3216600 Insertion of anal seton,

3216601 Adjustment of anal seton,

3216602 Removal of anal seton,

3211400 Per anal release of rectal stricture,

3214700 Incision of perianal thrombus,

3217400 Drainage of intra-anal abscess,

3217401 Drainage of perianal abscess,

3217402 Drainage of ischiorectal abscess,

9033800 Incision of rectum or anus,

3210500 Per anal full thickness excision of anorectal lesion or tissue,

3210800 Transsphincteric excision of lesion or tissue of rectum,

3211100 Reduction of rectal mucosa for rectal prolapse,

3214201 Excision of anal polyp,

3203000 Rectosigmoidectomy with formation of stoma,

3203001 Laparoscopic rectosigmoidectomy with formation of stoma,

3203900 Abdominoperineal proctectomy,

3204700 Perineal proctectomy,

3206000 Restorative proctectomy,

3211200 Perineal rectosigmoidectomy,

4399301 Definitive intestinal resection and pull-through anastomosis,

3202400 High anterior resection of rectum,

3202500 Low anterior resection of rectum,

3202600 Ultra low anterior resection of rectum,

3202800 Ultra low anterior resection of rectum with hand sutured coloanal anastomosis, 9220800 Anterior resection of rectum, level unspecified,

3201500 Total proctocolectomy with ileostomy,

3205100 Total proctocolectomy with ileo-anal anastomosis,

3205101 Total proctocolectomy with ileo-anal anastomosis and formation of temporary ileostomy,

3215900 Excision of anal fistula involving lower half of anal sphincter mechanism,

3215901 Insertion of seton for anal fistula involving lower half of anal sphincter mechanism, 3215902 Insertion of seton and excision of anal fistula involving lower half of anal sphincter mechanism,

3216200 Excision of anal fistula involving upper half of anal sphincter mechanism,

3216201 Insertion of seton for anal fistula involving upper half of anal sphincter mechanism, 3216202 Insertion of seton and excision of anal fistula involving upper half of anal sphincter mechanism,

3212300 Anoplasty,

4396300 Anorectoplasty via perineal approach,

4396600 Anorectoplasty via laparotomy,

3211700 Abdominal rectopexy,

3212600 Sphincteroplasty,

3213100 Perineal repair of rectocele,

3215300 Dilation of anus,

3216500 Repair of anal fistula with rectal mucosa advancement flap,

3220600 Insertion of gracilis neosphincter pacemaker,

3222100 Revision of artificial bowel sphincter,

9031300 Other repair of rectum,

9034200 Suture of laceration of rectum,

9034201 Suture of laceration of anus,

3213200 Administration of sclerosing agent for haemorrhoids,

3213500 Rubber band ligation of haemorrhoids,

3213501 Destruction of haemorrhoids,

3213800 Haemorrhoidectomy,

3213802 Stapled haemorrhoidectomy,

9031400 Other procedures on rectum,

9031600 Other procedures on anus,

9032801 Excision of lesion of peritoneal tissue with intestinal resection

**UPPER GASTROINTESTINAL SURGERY**

3053200 Oesophagogastric myotomy, laparoscopic approach,

3029302 Oesophagotomy,

3054500 Oesophagectomy by abdominal and thoracic mobilisation with thoracic anastomosis, large intestine interposition and anastomosis,

3054501 Oesophagectomy by abdominal and thoracic mobilisation with thoracic anastomosis using Roux-en-Y reconstruction,

3055001 Oesophagectomy by abdominal and thoracic mobilisation with cervical anastomosis using Roux-en-Y reconstruction,

3054100 Trans-hiatal oesophagectomy by abdominal and cervical mobilisation, with oesophagogastric anastomosis,

3054101 Trans-hiatal oesophagectomy by abdominal and cervical mobilisation, with oesophagojejunal anastomosis,

3053500 Oesophagectomy by abdominal and transthoracic mobilisation, with thoracic oesophagogastric anastomosis,

3053600 Oesophagectomy by abdominal and transthoracic mobilisation, with cervical oesophagogastric anastomosis,

3053601 Oesophagectomy by abdominal and transthoracic mobilisation, with cervical oesophagostomy,

3053304 Oesophagogastric myotomy, laparoscopic approach with fundoplasty,

3053305 Oesophagogastric myotomy, laparoscopic approach, with fundoplasty and closure of diaphragmatic hiatus,

3053300 Oesophagogastric myotomy, abdominal approach, with fundoplasty,

4384300 Repair of oesophageal atresia,

4384301 Repair of oesophageal atresia with repair of distal tracheo-oesophageal fistula, 4384302 Repair of oesophageal atresia with repair of proximal or multiple tracheo-oesophageal fistula,

3029300 Oesophagostomy,

3056000 Repair of oesophageal perforation,

3060600 Oesophageal transection via stapler,

9095400 Other reconstruction of oesophagus,

3037506 Gastrotomy,

3037515Gastrotomy with removal of foreign body,

3051800 Partial distal gastrectomy with gastroduodenal anastomosis,

3051801 Partial distal gastrectomy with gastrojejunal anastomosis,

3051802 Partial proximal gastrectomy with oesophagogastric anastomosis,

3050302 Partial gastrectomy with Roux-en-Y reconstruction following previous procedure for peptic ulcer disease,

3049702 Selective vagotomy with partial gastrectomy and Roux-en-Y reconstruction, 3052100 Total gastrectomy,

3052300 Subtotal gastrectomy,

3052400 Radical gastrectomy,

3050900 Control of bleeding peptic ulcer by gastric resection,

3037507 Gastrostomy,

3037531 Gastro-gastrostomy,

3051500 Gastro-enterostomy,

3037513 Pyloroplasty,

3052700 Fundoplasty, laparoscopic approach,

3052701 Fundoplasty, laparoscopic approach, with closure of diaphragmatic hiatus,

3052702 Fundoplasty, abdominal approach,

3052703 Fundoplasty, abdominal approach, with closure of diaphragmatic hiatus,

3052704 Fundoplasty, transthoracic approach,

3053000 Fundoplasty with cardiopexy,

3146600 Revision fundoplasty,

9034202 Suture of laceration of stomach,

3037510 Suture of perforated ulcer,

3037512 Reduction of gastric volvulus,

9030400 Other repair of stomach,

3051700 Reconstruction of pyloroplasty,

3051701 Reconstruction of gastroenterostomy

**HERNIA SURGERY**

3060902 Laparoscopic repair of inguinal hernia,

3060903Laparoscopic repair of inguinal hernia, bilateral,

3061402Repair of inguinal hernia, unilateral,

3061403Repair of inguinal hernia, bilateral,

3060900Laparoscopic repair of femoral hernia, unilateral,

3060901Laparoscopic repair of femoral hernia, bilateral,

3061400 Repair of femoral hernia, unilateral,

3061401 Repair of femoral hernia, bilateral,

3061700 Laparoscopic repair of femoral hernia, bilateral,

3061701 Repair of epigastric hernia,

3061702 Repair of linea alba hernia,

3040300 Repair of incisional hernia,

3040500 Repair of incisional hernia with muscle transposition,

3040501 Repair of incisional hernia with prosthesis,

3040502 Repair of incisional hernia with resection of strangulated intestine,

3056302 Repair of parastomal hernia,

3056303 Repair of parastomal hernia with resiting of stoma,

3040301 Repair of other abdominal wall hernia,

3040503 Repair of other abdominal wall hernia with muscle transposition,

3040504 Repair of other abdominal wall hernia with prosthesis,

3040505 Repair of incarcerated, obstructed or strangulated hernia,

3061500 Repair of traumatic diaphragmatic hernia,

3060000 Repair of traumatic diaphragmatic hernia,

3060100 Repair of diaphragmatic hernia with use of body wall flap or insertion of prosthetic patch,

3060101 Repair of diaphragmatic hernia, thoracic approach,

4383702 Repair of diaphragmatic hernia with use of body wall flap or insertion of prosthetic patch,

3017800 Closure of secondary defect of abdomen with umbilicus reconstruction

**CHOLECYSTECTOMIES**

3044300 Cholecystectomy,

3044500 Laparoscopic cholecystectomy,

3044800 Laparoscopic cholecystectomy with exploration of common bile duct via cystic duct,

3044900 Laparoscopic cholecystectomy with exploration of common bile duct via laparoscopic choledochotomy,

3045401 Cholecystectomy with choledochotomy,

3045500 Cholecystectomy with choledochotomy and biliary intestinal anastomosis,

9032000 Other repair of gallbladder

9034203 Repair of laceration of gall bladder

**APPENDICECTOMIES**

3057100 Appendicectomy,

3057200 Laparoscopic appendicectomy,

3037530 Appendicostomy,

9031100 Other procedures on appendix

**Supplementary Table 3:** ICD-10AM codes for MACE events

**MYOCARDIAL INFARCTION**

I210 Acute transmural myocardial infarction of anterior wall

I21 Acute myocardial infarction

I211 Acute transmural myocardial infarction of inferior wall

I212 Acute transmural myocardial infarction of other sites

I213 Acute transmural myocardial infarction of unspecified site

I214 Acute subendocardial myocardial infarction

I219 Acute myocardial infarction, unspecified

I22 Subsequent myocardial infarction

I220 Subsequent myocardial infarction of anterior wall

I221 Subsequent myocardial infarction of inferior wall

I228 Subsequent myocardial infarction of other sites

I229 Subsequent myocardial infarction of unspecified site

I23 Certain current complications following acute myocardial infarction

I230 Haemopericardium as current complication following acute myocardial infarction

I231 Atrial septal defect as current complication following acute myocardial infarction

I232 Ventricular septal defect as current complication following acute myocardial infarction

I233 Rupture of cardiac wall without haemopericardium as current complication following acute myocardial infarction

I234 Rupture of chordae tendineae as current complication following acute myocardial infarctionv

I235 Rupture of papillary muscle as current complication following acute myocardial infarction

I236 Thrombosis of atrium, auricular appendage, and ventricle as current complications following acute myocardial infarction

I238 Other current complications following acute myocardial infarction

I24 Other acute ischaemic heart diseases

I240 Coronary thrombosis not resulting in myocardial infarction

I241 Dressler's syndrome

I248 Other forms of acute ischaemic heart disease

I249 Acute ischaemic heart disease, unspecified

**CARDIAC ARREST**

I461 Sudden cardiac death, so described

I469 Cardiac arrest, unspecified

I460 Cardiac arrest with successful resuscitation

I46 Cardiac arrest

**CEREBROVASCULAR ACCIDENT**

I630 Cerebral infarction due to thrombosis of precerebral arteries

I631 Cerebral infarction due to embolism of precerebral arteries

I632 Cerebral infarction due to unspecified occlusion or stenosis of precerebral arteries

I633 Cerebral infarction due to thrombosis of cerebral arteries

I634 Cerebral infarction due to embolism of cerebral arteries

I635 Cerebral infarction due to unspecified occlusion or stenosis of cerebral arteries

I636 Cerebral infarction due to cerebral venous thrombosis, nonpyogenic

I638 Other cerebral infarction

I639 Cerebral infarction, unspecified

I63 Cerebral infarction

I610 Intracerebral haemorrhage in hemisphere, subcortical

I611 Intracerebral haemorrhage in hemisphere, cortical

I612 Intracerebral haemorrhage in hemisphere, unspecified

I613 Intracerebral haemorrhage in brain stem

I614 Intracerebral haemorrhage in cerebellum

I615 Intracerebral haemorrhage, intraventricular

I616 Intracerebral haemorrhage, multiple localized

I618 Other intracerebral haemorrhage

I619 Intracerebral haemorrhage, unspecified

I620 Nontraumatic subdural haemorrhage

I621 Nontraumatic extradural haemorrhage

I629 Intracranial haemorrhage (nontraumatic), unspecified

I600 Subarachnoid haemorrhage from carotid siphon and bifurcation

I601 Subarachnoid haemorrhage from middle cerebral artery

I602 Subarachnoid haemorrhage from anterior communicating artery

I603 Subarachnoid haemorrhage from posterior communicating artery

I604 Subarachnoid haemorrhage from basilar artery

I605 Subarachnoid haemorrhage from vertebral artery

I606 Subarachnoid haemorrhage from other intracranial arteries

I607 Subarachnoid haemorrhage from intracranial artery, unspecified

I608 Other subarachnoid haemorrhage

I609 Subarachnoid haemorrhage, unspecified

G463 Brain stem stroke syndrome

I64 Stroke, not specified as haemorrhage or infarction

I694 Sequelae of stroke, not specified as haemorrhage or infarction

**Supplementary Table 4:** Types of surgery (Elective vs. Emergency procedures)

|  | Elective | Emergency |
| --- | --- | --- |
| Bowel surgery (large and small) | 1,152 | 1,194 |
| Anorectal surgery | 780 | 594 |
| Upper GI | 73 | 102 |
| Hernia surgery | 2,871 | 913 |
| Cholecystectomy | 154 | 156 |
| Appendicectomies | - | 412 |

**Supplementary Figure 1A-E:** Calibration plots: Observed risk of MACE plotted against the predicted. Solid line represented perfect calibration. Solid dots represent grouping of predicted risks. Grouped estimates are below the dashed line suggestive over overestimation of risk.

Figure 1A: Calibration plot (all elective surgery <65).

Figure 1B: Calibration plot (all elective surgery ≥65).

Figure 1C: Calibration plot (all elective surgery in transplant patient only).

Figure 1D: Calibration plot (all elective surgery in dialysis patient only).

Figure 1E: Calibration plot (all emergency surgeries only).

**Supplementary figure 2:** Decision curve analysis (all elective surgeries) plotting net benefit against threshold probability, comparing the clinical usefulness of RCRI versus strategies of all patients having surgery (green dashed line) and no participants having surgery (solid red line).

Figure 2A: Decision curve analysis (all elective surgeries <65).

Figure 2B: Decision curve analysis (all elective surgeries ≥65).

Figure 2C: Decision curve analysis (all elective surgeries in transplant patients only).

Figure 2D: Decision curve analysis (all elective surgeries in transplant patients only).

Figure 2E: Decision curve analysis (all emergency surgeries).
